# Author Correction: Impact of delivery mode-associated gut microbiota dynamics on health in the first year of life

**DOI:** 10.1038/s41467-019-13373-1

**Published:** 2019-11-25

**Authors:** Marta Reyman, Marlies A. van Houten, Debbie van Baarle, Astrid A. T. M. Bosch, Wing Ho Man, Mei Ling J. N. Chu, Kayleigh Arp, Rebecca L. Watson, Elisabeth A. M. Sanders, Susana Fuentes, Debby Bogaert

**Affiliations:** 1 Department of Paediatric Immunology and Infectious Diseases, Wilhelmina Children’s Hospital of University Medical Centre, Utrecht, the Netherlands; 2 Spaarne Gasthuis Academy Hoofddorp and Haarlem, Hoofddorp, The Netherlands; 3 National Institute for Public Health and the Environment, Bilthoven, The Netherlands; 4 Medical Research Council/University of Edinburgh Centre for Inflammation Research, Queen’s Medical Research Institute, University of Edinburgh, Edinburgh, UK

**Keywords:** Microbiome, Infectious diseases, Paediatric research

Correction to: *Nature Communications* 10.1038/s41467-019-13014-7, published online 01 November 2019.

The original version of this Article contained an error in the number of unique bacterial taxa identified by whole genome shotgun sequencing, which was incorrectly given as 137 instead of 119. This has been corrected in both the PDF and HTML versions of the Article.

