# Supplementary material for: Impact of delivery mode-associated gut microbiota dynamics on health in the first year of life
Source: Nat Commun. 2019 Nov 1;10:4997. doi: 10.1038/s41467-019-13014-7 (PMC6825150; doi:10.1038/s41467-019-13014-7)
Supplement: Supplementary file 1 — Supplementary Information [file 41467_2019_13014_MOESM1_ESM.pdf]

SUPPLEMENTARY INFORMATION

Reyman *et al.* Impact of delivery mode-associated gut microbiota dynamics on health in the first year of life.

Supplementary Figures 1

Supplementary Figure 1: Flowchart showing number of samples aimed for, obtained, and of sufficient quality for analyses 2

Supplementary Figure 2: Diversity indices 3

Supplementary Figure 3: Associations between baseline characteristics and fecal microbiota composition 4

Supplementary Figure 4: NMDS plot of mothers' samples stratified by delivery mode 5

Supplementary Figure 5: Comparison of 16S rRNA OTUs with species found by WGS sequencing 6

Supplementary Figure 6: NMDS plot of samples analyzed by WGS sequencing 7

Supplementary Figure 7: Statistical analysis scheme 8

Supplementary Figure 8: NMDS plot of all samples colored by specific delivery mode 11

Supplementary Tables 12

Supplementary Table 1: Decrease in BC dissimilarity between children's and mothers' samples over time 12

Supplementary Table 2: Effect of delivery mode on overall gut microbiota composition in exclusively formula fed children 13

Supplementary Table 3: FitTimeSeries results of differentially abundant taxa between delivery mode groups 14

Supplementary Table 4: Random forest validation of biomarker species found at 1 week of life associated with RI events 19

Supplementary Table 5: Correlation between top 5 most abundant 16S rRNA OTUs and WGS species 20

Supplementary Table 6: Confirmation of significant differences in E. coli, Klebsiella spp. and Enterococcus spp. between delivery mode groups by qPCR 21

Supplementary Table 7: Confirmation of association between Klebsiella spp. and Enterococcus spp. colonization at 1 week of life and more RI events later in life 22

Supplementary Figures

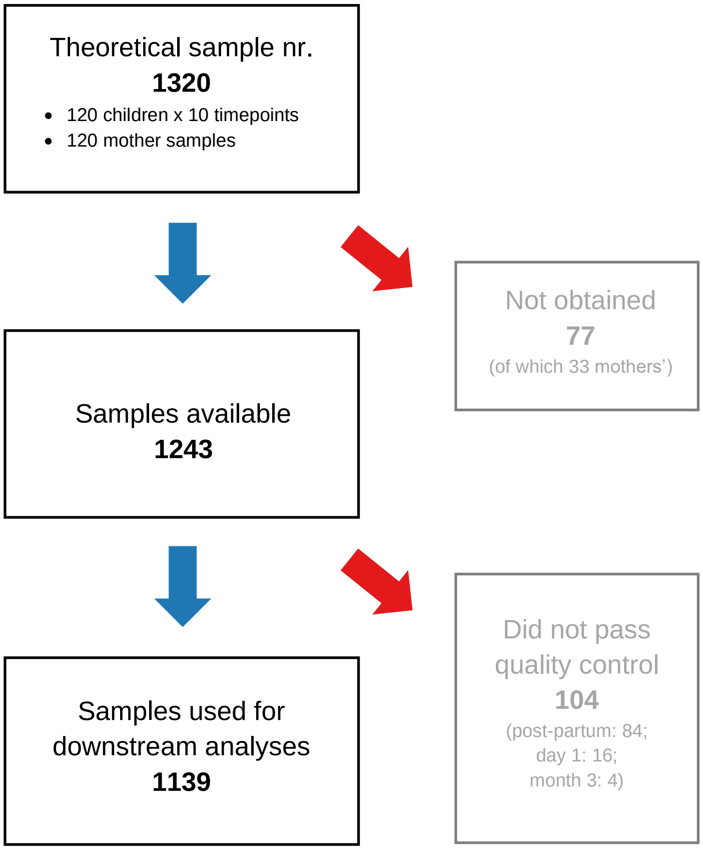

Supplementary Figure 1: Flowchart showing number of samples aimed for, obtained, and of sufficient quality for analyses.

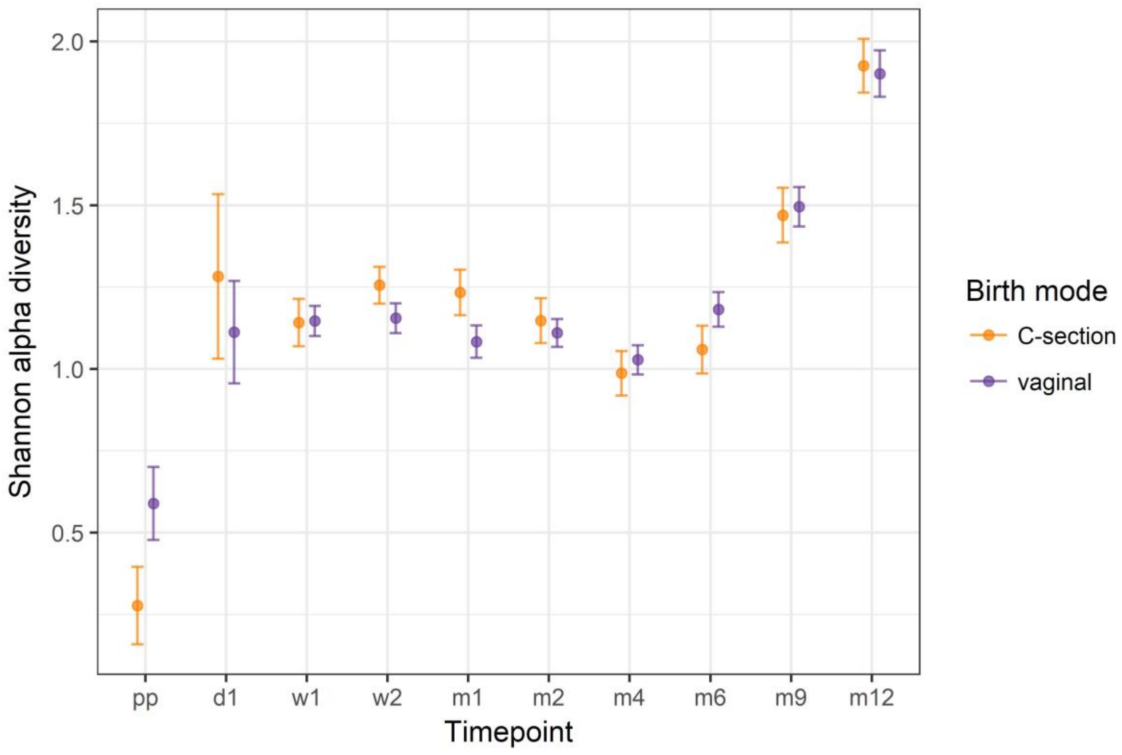

Supplementary Figure 2: Diversity indices. Differences in Shannon alpha diversity of the fecal microbiota between children born vaginally or by C-section plotted per timepoint and of the mother samples obtained two weeks after delivery. The data points represent the means per group per timepoint and the error bars represent the standard errors of the means (+/- 1SD). There were no significant differences found in alpha diversity between the two delivery mode groups at any timepoint. Source data are provided as a Source Data file.

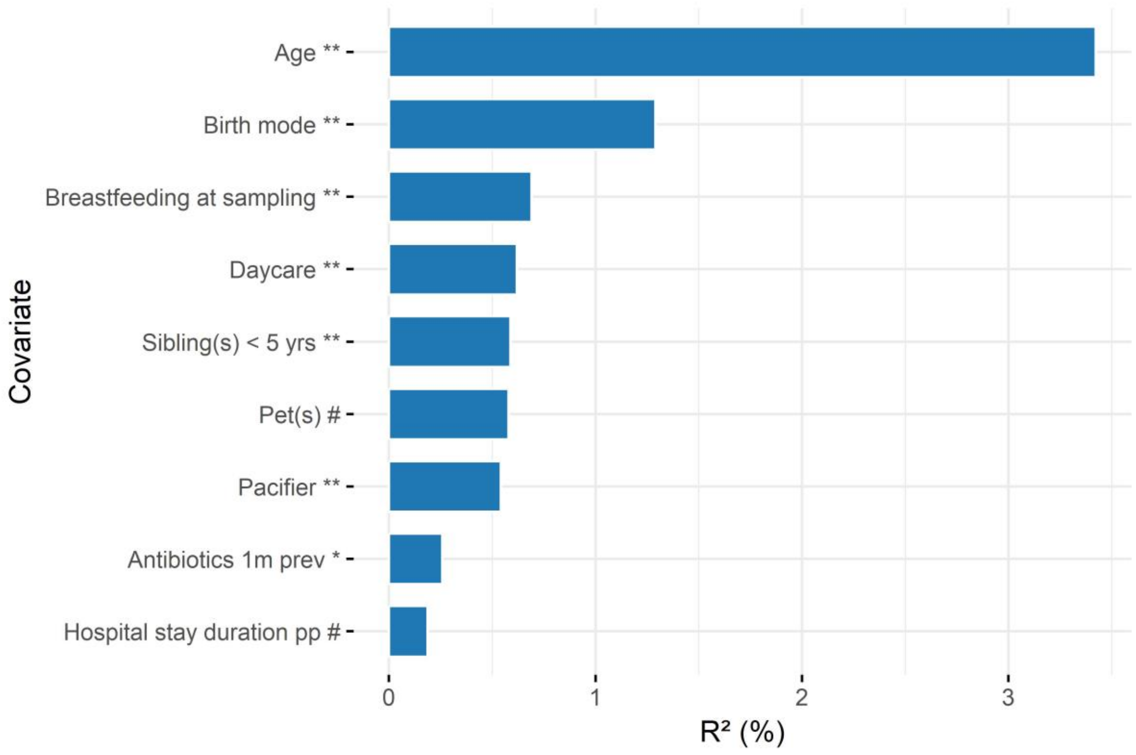

Supplementary Figure 3: Associations between baseline characteristics and fecal microbiota composition. Covariates are shown that were significantly correlated with fecal microbiota at any timepoint in univariate analyses were included in a multivariate permutational multivariate analysis of variance (PERMANOVA)-test over all timepoints using 1999 permutations with the strata parameter set to participant. The percentage of variance explained (R^2 (%)) is plotted on the x-axis and # = adjusted p-value <0.1 (in both cases 0.061), * = adjusted p-value <0.05 and ** = adjusted p-value <0.01.

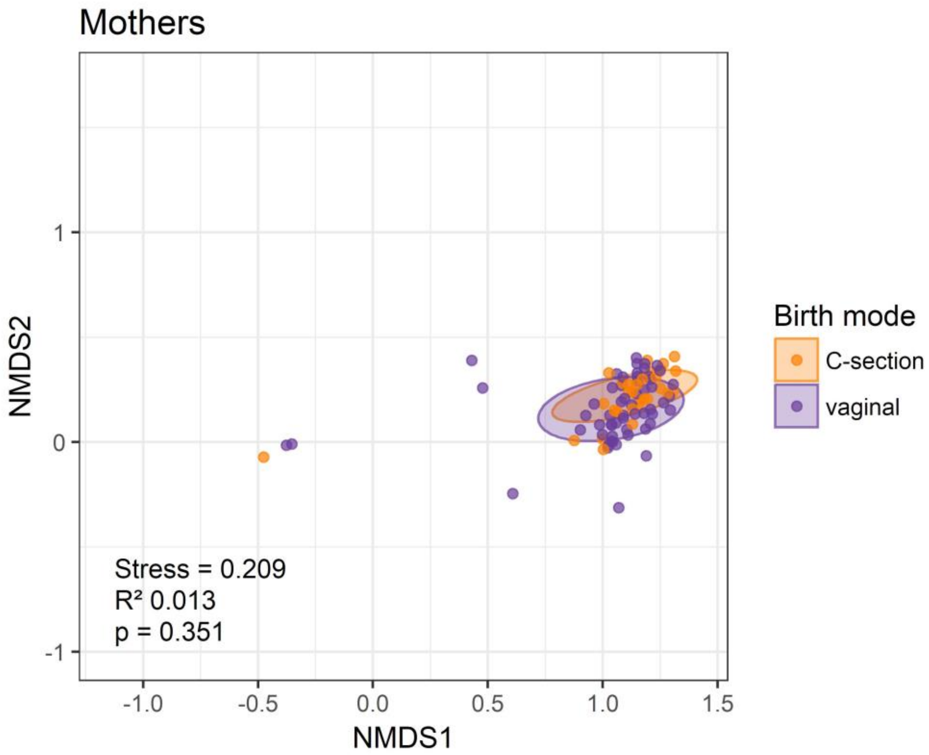

Supplementary Figure 4: NMDS plot of mothers' samples stratified by delivery mode. Non-metric multidimensional scaling (nMDS) plot visualizing the overall gut bacterial community composition of the mothers' samples collected two weeks after delivery. Each data point represents the microbial community composition of one sample. The ellipses represent the standard deviation of data points belonging to one of two groups: mothers that gave birth vaginally (purple; n=57 biologically independent samples) or by C-section (orange; n=30 biologically independent samples). The center points of the ellipses were calculated using the mean of the coordinates of each group. The stress, effect size (R^2) calculated by multivariate permutational multivariate analysis of variance (PERMANOVA)-test and corresponding p-value are shown in the plot. Even though all mothers that delivered by C-section received antibiotics after the clamping of the umbilical cord, their overall but bacterial community composition 2 weeks after delivery does not differ from mothers that gave birth vaginally and did not receive post-partum antibiotics. Source data are provided as a Source Data file.

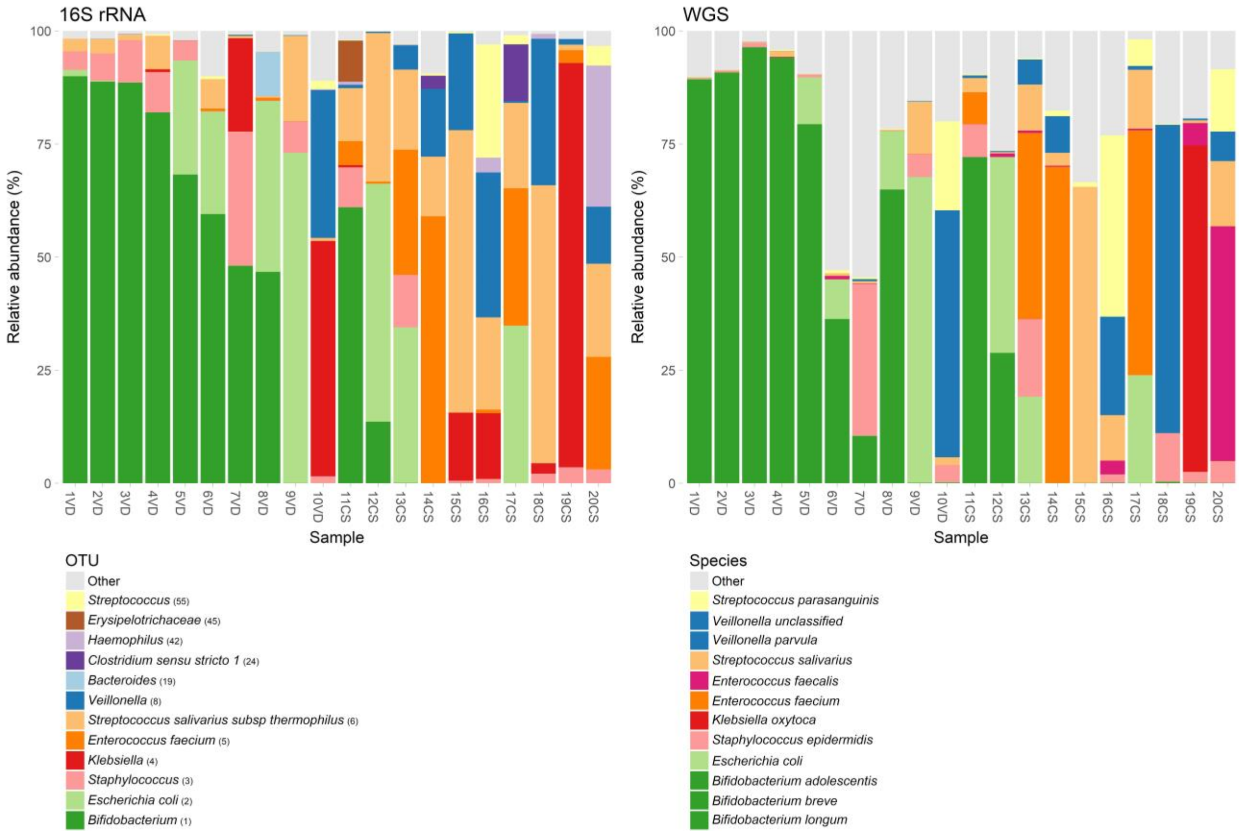

Supplementary Figure 5: Comparison of 16S rRNA OTUs with species found by WGS sequencing. A random subset of 20 samples were analyzed by whole genome shotgun (WGS) sequencing. The mean relative abundances of the 12 most abundant 16S rRNA OTUs were compared to the species found by WGS sequencing. Taxa approaching a similar annotation in both methods are colored similarly to allow for easier visual comparison. VD = vaginal delivery, CS = caesarean section. Source data are provided as a Source Data file.

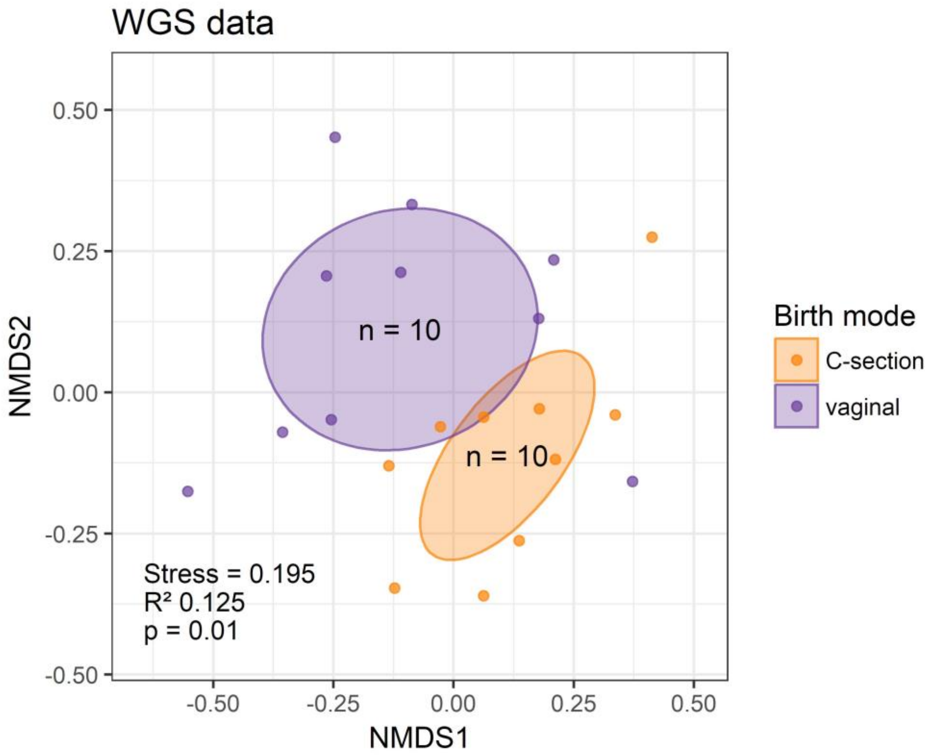

Supplementary Figure 6: NMDS plot of samples analyzed by WGS sequencing. Non-metric multidimensional scaling (nMDS) plot visualizing the differences in overall gut microbial community composition between the delivery mode groups at 1 week of age using the whole genome shotgun (WGS) sequencing data of a randomly selected subset of 20 samples (n represents the number of biologically independent samples per group). Each data point represents the microbial community composition of one sample. The ellipses represent the standard deviation of data points belonging to each delivery mode group, with the center points of the ellipses calculated using the mean of the coordinates per group. The stress of the ordination, number of children per group, effect size (R^2) calculated by multivariate permutational multivariate analysis of variance (PERMANOVA)-test and corresponding p-value are printed in the plot. Source data are provided as a Source Data file.

Flow in data analyses (with type of test(s) in italic) to address the following research questions:

<u>Primary research question</u>

Are there differences in gut microbiota development between delivery mode groups?

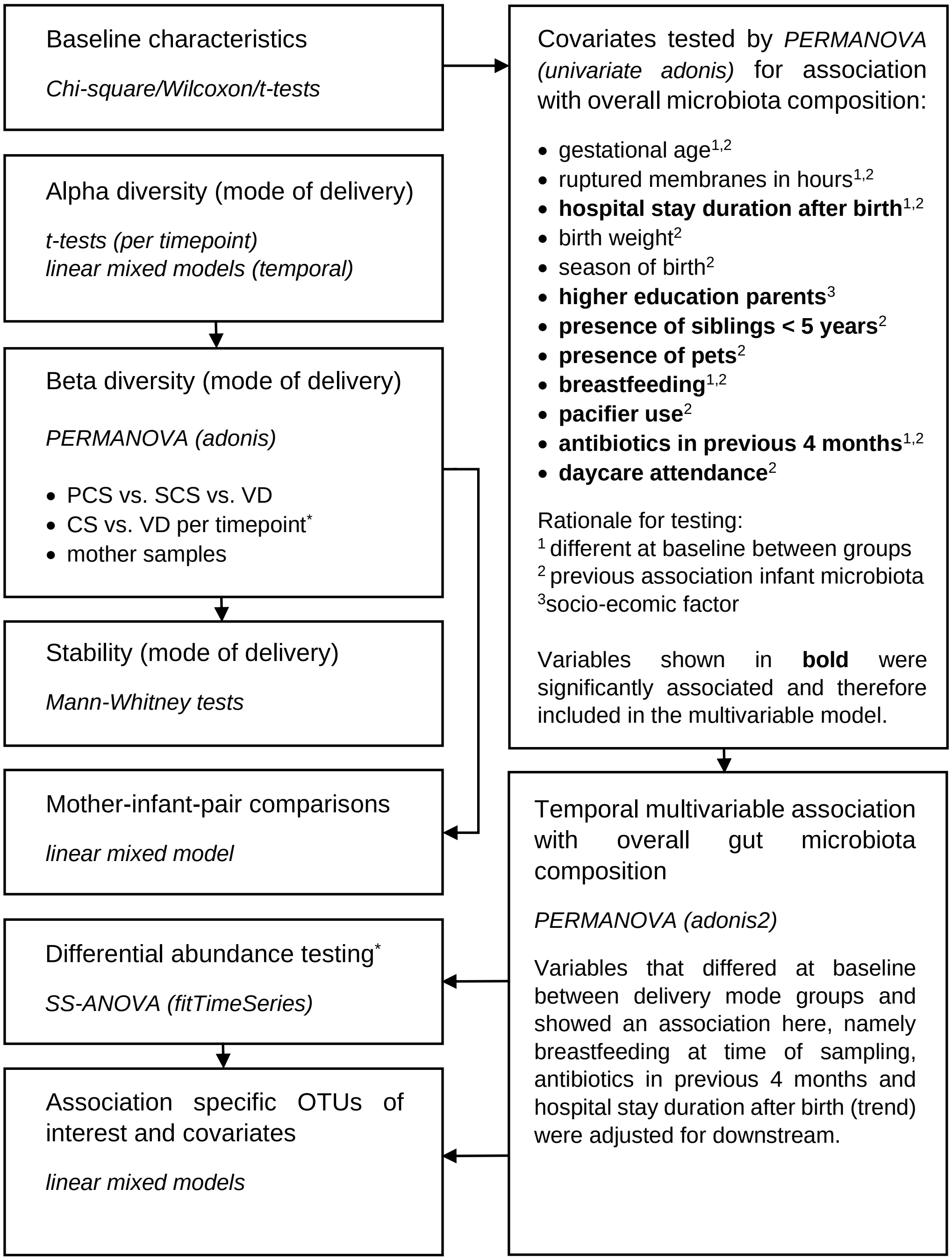

* Post hoc testing of differences in beta diversity and differential abundance testing of taxa for subset of children receiving exclusive formula feeding to exclude potential confounding of antibiotic transmission through breastfeeding on delivery-mode related microbiota findings. PCS = primary caesarean section (CS), SCS = secondary CS, VD = vaginally delivered, OTU = operational taxonomical unit

<u>Secondary research question</u>

1. Is gut microbiota composition in early life associated with health?

Association between gut microbiota composition at 1 week (max. differences observed for mode of delivery effect) and health outcomes later in life for:

- fever
- **categorized number of RI events**
- gastrointestinal (GI) symptoms
  - post partum nausea
  - constipation
  - diarrhea
  - vomiting
  - GP visits for GI symptoms
  - specialist visits for GI symptoms
- thrush
- number of antibiotic courses

*PERMANOVA (adonis)**

Variable shown in **bold** was significantly associated and therefore further tested in association with biomarker species.

→

Assessment of OTUs driving the association between gut microbiota composition at 1 week of life and categorized number of RI events

*fitZig**

↓

Validation of fitZig results*

random forest analysis

* Post-hoc analyses on stratified data per delivery mode group to exclude potential confounding effect of delivery mode group on outcome. RI = respiratory infection

Exploratory research questions

Can 16S rRNA sequencing taxonomical annotation be validated with whole genome shotgun (WGS) sequencing and can the differences in biomarkers between delivery mode groups found by 16S rRNA be confirmed by WGS and qPCR?

Correlation 16S rRNA and WGS results (subset of 20 week 1 samples)

Pearson correlation tests

→

Differences in abundance of species in WGS dataset between delivery mode groups/differences in species presence identified by qPCR between delivery mode groups and RI event categories

Wilcoxon tests/chi-square tests

Exploratory (post hoc) analyses

Is delivery mode associated with health characteristics?

Group differences in health characteristics

Chi-square tests

→

Comparison of hazard ratio for antibiotic prescription between delivery mode groups

Cox proportional hazard model

Supplementary Figure 7: Statistical analysis scheme.

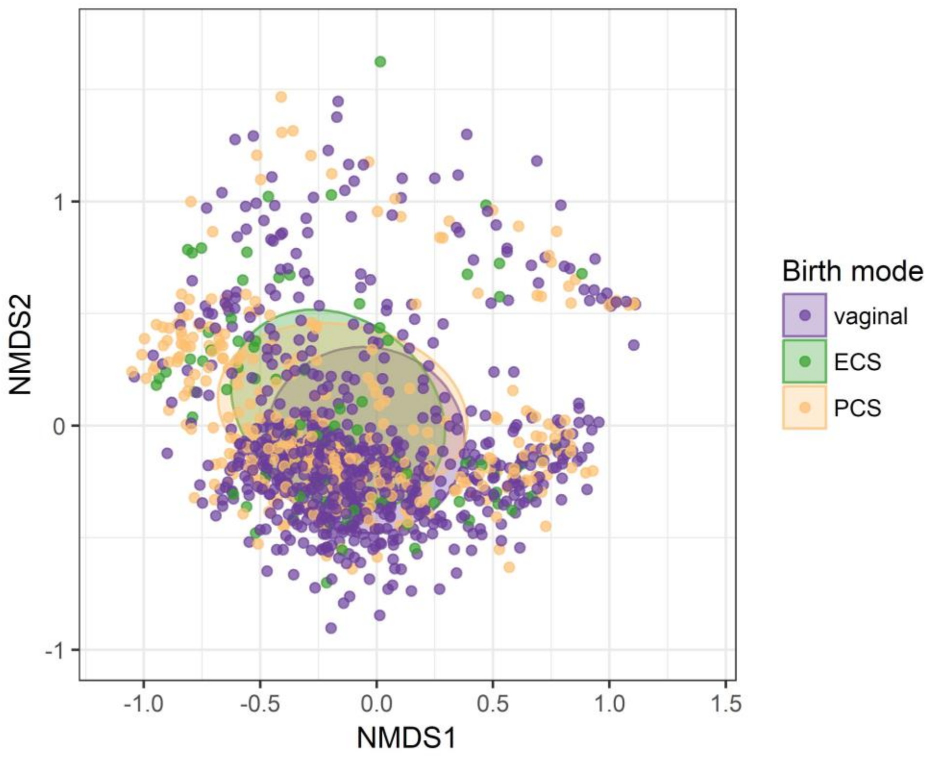

Supplementary Figure 8: NMDS plot of all samples colored by specific delivery mode. Non-metric multidimensional scaling (nMDS) plot visualizing the overall gut bacterial community composition of all participants' samples colored by delivery mode, specified as vaginal, emergency CS (ECS) or planned CS (PCS) delivery. Each data point represents the microbial community composition of one sample. The ellipses represent the standard deviation of data points belonging to each group. The overall gut bacterial community composition of children born by ECS (n=10) was more similar to that of children born by PCS (permutational multivariate analysis of variance [PERMANOVA]-test, R^2 0.005, p=0.051) than by VD (R^2 0.006, p=0.002), hence the ECS and PCS children were grouped together for delivery mode comparisons. Source data are provided as a Source Data file.

Supplementary Tables

Timepoint	median BC dissimilarity	IQR25	IQR75
Post-partum	0.999	0.996	1.000
Day 1	0.986	0.903	0.998
Week 1	0.982	0.922	0.996
Week 2	0.971	0.895	0.993
Month 1	0.954	0.864	0.985
Month 2	0.935	0.845	0.978
Month 4	0.923	0.837	0.971
Month 6	0.904	0.807	0.966
Month 9	0.834	0.725	0.924
Month 12	0.739	0.633	0.839

Supplementary Table 1: Decrease in BC dissimilarity between children's and mothers' samples over time. Overview of groupwise Bray-Curtis (BC) dissimilarity indices between children's and mothers' samples per timepoint. IQR = interquartile range. Source data are provided as a Source Data file.

Timepoint	R^2	Adjusted p-value
Day 1	0.082	0.441
Week 1	0.215	0.008
Week 2	0.152	0.044
Month 1	0.038	1.000
Month 2	0.029	1.000

Supplementary Table 2: Effect of delivery mode on overall gut microbiota composition in exclusively formula fed children. Effect sizes (R^2) calculated by multivariate permutational multivariate analysis of variance (PERMANOVA)-tests and corresponding adjusted p-values are shown for the effect of delivery mode on overall gut microbiota composition of the exclusively formula fed subset of participants. The results for the post-partum timepoint are not shown because there was only one sample available per group. The period until 2 months was studied as this was were the biggest differences in microbiota were found for the overall cohort.

	OTU	Interval nr.	Interval start	Interval end	Area	Adjusted p-value
1	*Bifidobacterium* (1)	interval:1	1	30	112,712	0,003287
2	*Escherichia coli* (2)	interval:1	0	85	167,6269	0,003287
3	*Klebsiella* (4)	interval:1	0	139	-276,087	0,003287
4	*Enterococcus faecium* (5)	interval:1	7	35	-65,1366	0,003287
5	*Enterococcus faecium* (5)	interval:2	269	361	133,5926	0,016636
6	*Enterococcus faecium* (5)	interval:3	362	368	10,49423	0,003287
7	*Streptococcus salivarius subsp thermophilus* (6)	interval:2	0	25	-21,0815	0,011449
8	*Veillonella* (8)	interval:1	0	73	-100,675	0,003287
9	*Bifidobacterium bifidum* (11)	interval:1	0	68	79,26634	0,003287
10	*ratAN060301C* (12)	interval:1	0	180	191,876	0,003287
11	*Pseudobutyrivibrio* (13)	interval:1	360	379	23,58741	0,045462
12	*Bifidobacterium dentium* (14)	interval:1	0	1	0,81083	0,003287
13	*Bifidobacterium dentium* (14)	interval:2	43	74	-28,6032	0,009705
14	*Bifidobacterium dentium* (14)	interval:3	247	282	40,26375	0,003287
15	*Collinsella* (16)	interval:1	0	170	125,4543	0,00794
16	*Faecalibacterium* (17)	interval:1	0	10	-8,48254	0,04458
17	*Faecalibacterium* (17)	interval:2	203	354	-252,214	0,039765
18	*Faecalibacterium* (17)	interval:3	355	378	-34,3762	0,03537
19	*Streptococcus gallolyticus subsp macedonicus* (18)	interval:1	0	353	520,936	0,003287
20	*Bacteroides* (19)	interval:1	0	303	683,5567	0,003287
21	*Clostridium sensu stricto 1* (21)	interval:1	0	191	-204,811	0,003287
22	*Lactobacillus* (23)	interval:1	0	184	-106,637	0,045462
23	*Clostridium sensu stricto 1* (24)	interval:1	0	146	-107,426	0,003287
24	*Veillonella* (27)	interval:1	0	385	458,6269	0,003287
25	*Clostridium butyricum* (29)	interval:1	231	385	92,90985	0,003287
26	*Lachnospiraceae* (30)	interval:1	0	103	-43,8028	0,009705
27	*Bacteroides* (35)	interval:1	0	374	513,9418	0,003287
28	*Peptostreptococcaceae* (36)	interval:1	0	308	-208,9	0,003287
29	*Peptostreptococcaceae* (37)	interval:1	17	211	-62,1764	0,02857
30	*Eubacterium hallii* (39)	interval:1	365	385	50,32886	0,047501
31	*Lactobacillus acidophilus* (40)	interval:1	0	111	40,92728	0,003287

32	*Bacteroides* (48)	interval:1	29	272	179,8846	0,003287
33	*Bacteroides* (53)	interval:1	0	241	97,51382	0,003287
34	*Dorea* (54)	interval:1	42	263	-144,523	0,003287
35	*Streptococcus* (55)	interval:1	0	57	-43,929	0,003287
36	*Streptococcus* (55)	interval:2	169	259	50,76685	0,005661
37	*Proteus mirabilis* (63)	interval:1	0	59	10,77698	0,003287
38	*Bacteroides* (65)	interval:1	8	317	184,1286	0,003287
39	*Lachnospiraceae* (68)	interval:1	248	385	35,62635	0,016636
40	*Streptococcus* (69)	interval:1	0	77	-35,2993	0,021978
41	*Lactobacillus fermentum* (75)	interval:1	68	385	171,5	0,003287
42	*Ruminococcaceae* (76)	interval:1	268	324	-60,6412	0,015506
43	*Ruminococcaceae* (76)	interval:2	362	385	39,14089	0,003287
44	*Roseburia* (77)	interval:1	253	280	-27,9272	0,003287
45	*Roseburia* (77)	interval:2	325	363	53,83287	0,031899
46	*Coprococcus* (92)	interval:1	365	385	19,34755	0,029643
47	*Clostridium sensu stricto* (95)	interval:1	58	385	-75,2924	0,02857
48	*Lachnospiraceae* (101)	interval:1	328	385	-25,165	0,046671
49	*Eubacterium desmolans* (106)	interval:1	214	385	-121,624	0,003287
50	*Rothia* (113)	interval:1	0	59	-40,3232	0,003287
51	*Rothia* (113)	interval:2	165	269	48,55794	0,003287
52	*Megasphaera* (122)	interval:1	14	42	-7,17491	0,00794
53	*Megasphaera* (122)	interval:2	223	360	75,28518	0,03675
54	*bacterium mpn isolate group 25* (124)	interval:1	370	385	15,47146	0,032815
55	*Lachnospiraceae* (127)	interval:1	29	274	-50,5619	0,03537
56	*Roseburia* (133)	interval:1	276	358	-98,0038	0,023743
57	*Roseburia* (133)	interval:2	367	385	25,386	0,011449
58	*Marvinbryantia* (137)	interval:1	18	26	1,600834	0,003287
59	*Ruminococcaceae* (138)	interval:1	15	24	2,685455	0,003287
60	*Bifidobacterium* (147)	interval:1	0	44	14,10129	0,003287
61	*Bifidobacterium* (147)	interval:2	282	385	-40,6182	0,011449
62	*Subdoligranulum* (148)	interval:1	177	385	-83,4036	0,03066
63	*Lachnospiraceae* (153)	interval:1	139	385	86,92777	0,021978
64	*Lachnospiraceae* (155)	interval:3	288	356	-74,418	0,042053
65	*Varibaculum* (161)	interval:1	10	59	-17,5114	0,005661

66	*Varibaculum* (161)	interval:2	118	154	13,81666	0,029643
67	*Blautia* (169)	interval:1	14	25	4,242241	0,003287
68	*Parasutterella* (171)	interval:1	75	385	102,9637	0,00794
69	*Bifidobacterium animalis* (175)	interval:1	0	153	-27,2805	0,00794
70	*Anaerosporobacter* (181)	interval:1	368	385	15,52433	0,03066
71	*Bifidobacterium* (182)	interval:1	42	195	-36,15	0,009705
72	*Escherichia Shigella* (185)	interval:1	0	41	13,9863	0,003287
73	*Sutterella* (187)	interval:1	49	259	37,64495	0,025232
74	*Streptococcus* (189)	interval:1	0	33	-8,99449	0,020585
75	*Streptococcus* (189)	interval:2	181	277	25,44483	0,003287
76	*Bifidobacteriaceae* (198)	interval:1	119	385	-90,3912	0,009705
77	*Desulfovibrio* (201)	interval:1	128	385	-48,1528	0,032815
78	*Finegoldia* (203)	interval:1	0	159	-32,6427	0,003287
79	*Bifidobacterium dentium* (205)	interval:1	120	385	-66,4652	0,016636
80	*Collinsella* (211)	interval:1	18	247	72,1091	0,049817
81	*Collinsella* (215)	interval:1	0	385	138,1499	0,003287
82	*Lachnospiraceae* (221)	interval:1	0	5	-1,23682	0,04458
83	*Lachnospiraceae* (221)	interval:2	268	271	0,732577	0,005661
84	*Lachnospiraceae* (221)	interval:3	323	361	-23,455	0,040759
85	*Lachnospiraceae* (221)	interval:4	376	385	6,209622	0,031899
86	*Bacteroides* (245)	interval:1	183	385	-62,2234	0,009705
87	*Bifidobacterium* (246)	interval:1	0	173	45,25739	0,003287
88	*Enterococcaceae* (251)	interval:1	0	41	-10,8802	0,013437
89	*Klebsiella* (252)	interval:1	0	113	-44,4969	0,003287
90	*Enterococcus* (256)	interval:1	0	70	-22,7148	0,003287
91	*Bifidobacterium breve* (257)	interval:1	0	369	205,9727	0,003287
92	*Bifidobacterium breve* (261)	interval:1	0	234	46,40682	0,011449
93	*Negativicoccus* (263)	interval:1	0	175	-32,338	0,003287
94	*Streptococcus* (268)	interval:1	0	385	143,3426	0,003287
95	*Anaerostipes* (271)	interval:1	0	2	0,232903	0,003287
96	*Veillonella* (275)	interval:1	0	46	-5,37729	0,047501
97	*Eubacterium hallii* (278)	interval:1	271	348	-39,4095	0,005661
98	*Eubacterium hallii* (278)	interval:2	365	385	14,2956	0,003287
99	*Bacteroides* (279)	interval:1	17	199	46,71601	0,003287

100	*Citrobacter sedlakii* (288)	interval:1	0	141	-18,3047	0,005661
101	*bacterium NLAE zl C423* (298)	interval:1	0	183	21,71035	0,003287
102	*Bifidobacteriaceae* (299)	interval:1	0	49	6,191564	0,003287
103	*bacterium NLAE zl C350* (303)	interval:1	0	39	5,321786	0,003287
104	*Bifidobacteriaceae* (309)	interval:1	346	385	9,008275	0,003287
105	*Bacteroides* (310)	interval:1	49	385	80,96046	0,016636
106	*Bifidobacterium* (320)	interval:1	0	95	10,17972	0,003287
107	*Bacteroides* (326)	interval:1	143	239	14,71285	0,016636
108	*Bacteroides fragilis* (327)	interval:1	0	374	149,0278	0,003287
109	*Coprobacillus* (331)	interval:1	43	167	-21,4177	0,005661
110	*Gardnerella* (333)	interval:1	105	385	52,21995	0,009705
111	*Peptostreptococcaceae* (335)	interval:1	65	223	-17,8886	0,040759
112	*Leuconostoc* (341)	interval:1	29	287	36,92326	0,003287
113	*Pseudobutyrivibrio* (349)	interval:1	91	385	45,94751	0,005661
114	*Veillonella* (366)	interval:1	142	168	-6,0963	0,034251
115	*Pasteurellaceae* (385)	interval:1	0	50	-5,49709	0,021978
116	*Alistipes* (388)	interval:1	120	385	-51,374	0,011449
117	*Aggregatibacter* (390)	interval:1	0	127	-15,8943	0,005661
118	*Odoribacter* (398)	interval:1	323	360	-21,2673	0,005661
119	*Odoribacter* (398)	interval:3	371	385	8,259985	0,003287
120	*Streptococcus gallolyticus subsp macedonicus* (399)	interval:1	0	310	71,0579	0,003287
121	*Bifidobacteriaceae* (430)	interval:1	34	152	-27,4874	0,009705
122	*Bacteroides* (433)	interval:1	0	129	12,50547	0,013437
123	*Bifidobacterium* (435)	interval:1	0	84	14,61852	0,003287
124	*bacterium NLAE zl G195* (451)	interval:1	0	192	23,93176	0,003287
125	*Actinomyces sp oral clone DR002* (463)	interval:1	22	188	-28,9075	0,016636
126	*Lactobacillus* (478)	interval:1	19	168	-30,5026	0,029643
127	*Bacteroides* (480)	interval:1	39	234	22,8699	0,00794
128	*Propionibacterium* (483)	interval:1	54	148	-8,69648	0,03066
129	*Anaerostipes* (488)	interval:1	238	301	-23,7484	0,025232
130	*Bacteroides* (491)	interval:1	0	287	38,98332	0,003287
131	*Lachnospiraceae* (496)	interval:1	116	202	-14,4411	0,032815
132	*Peptostreptococcaceae* (499)	interval:1	49	295	19,37888	0,005661

133	*Streptococcus* (502)	interval:1	0	85	-11,6154	0,003287
134	*Veillonella* (518)	interval:2	1	133	-18,0585	0,003287
135	*Staphylococcus* (530)	interval:1	0	24	-2,91257	0,048668

Supplementary Table 3: FitTimeSeries results of differentially abundant taxa between delivery mode groups. Differential abundance testing by smoothing spline analysis of variance (SS-ANOVA) was executed to test in which specific intervals significant differences in OTUs existed between the delivery mode groups, adjusted for duration of hospital stay after birth, breastfeeding at time of sampling and antibiotic use in the 4 weeks prior to sampling. A positive area value indicates that the abundance of a specific OTU is higher in the VD group, while a negative area value indicates that the abundance of an OTU is higher in the CS group. To correct for multiple testing, the Benjamini-Hochberg method was applied and only results with an adjusted p-values of <0.05 are shown.

OTU	Mean decrease in Gini coefficient
Bifidobacterium (1)	1.372
Enterococcus faecium (5)	1.300
Klebsiella (4)	1.190
Staphylococcus (3)	1.143
Streptococcus (55)	1.014
Haemophilus (42)	1.012
Streptococcus salivarius subsp thermophilus (6)	0.980
Streptococcus (189)	0.890
Bifidobacterium (435)	0.866
Rothia (113)	0.810

Supplementary Table 4: Random forest validation of biomarker species found at 1 week of life associated with RI events. Random forest analysis was performed on the samples collected at 1 week of life to validate the biomarker species found with fitZig analysis to be associated with respiratory infection (RI) events later in life. The categorized RI events (0-2 or 3-7) were set as outcome and the relative abundance of OTUs as predictors, together with delivery mode, duration of hospital stay after birth, feeding type and antibiotics in the 4 weeks prior to sampling. Biomarkers most discriminative of outcome are shown. Again, *Bifidobacterium*, *Enterococcus* and *Klebsiella* were found to be important taxa in the association between microbiota composition at 1 week of life and the number of RI events in the first year.

**16S rRNA OTUs**	**WGS species**	**Pearson's r**	**Adjusted p-value**
Bifidobacterium (1)	*Bifidobacterium longum*<br>*Bifidobacterium breve*<br>*Bifidobacterium adolescentis*	0.95	<0.001
Escherichia coli (2)	*Escherichia coli*	0.95	<0.001
Staphylococcus (3)	*Staphylococcus epidermidis*	0.86	<0.001
Klebsiella (4)	*Klebsiella oxytoca*	0.83	<0.001
Enterococcus faecium (5)	*Enterococcus faecium*	0.92	<0.001

Supplementary Table 5: Correlation between top 5 most abundant 16S rRNA OTUs and WGS species. The top 5 most abundant OTUs of the 16S rRNA dataset were correlated with the corresponding whole genome shotgun (WGS) sequencing species using Pearson correlations. The relative abundances of the 3 most abundant *Bifidobacterium* species of the WGS dataset were combined. An adjusted p-value of <0.05 stands for a significant correlation (not a significant difference). Source data are provided as a Source Data file.

**qPCR positive for:**	**Vaginal birth**	**C-section birth**	**p**
n (%)	74	46	
E. coli (%)	53 (71.6)	16 (36.4)	*<0.001*
Klebsiella spp. (%)	7 (9.5)	13 (29.5)	*0.011*
Enterococcus spp. (%)	40 (54.1)	36 (81.8)	*0.004*

Supplementary Table 6: Confirmation of significant differences in E. coli, Klebsiella spp. and Enterococcus spp. between delivery mode groups by qPCR. Quantitative polymerase chain reaction (qPCR) analysis was performed for *E. coli*, *Klebsiella* spp. and *Enterococcus* spp. on all samples of infants collected at 1 week of life (n=119). One sample was discarded from analysis due to an abnormal signal of its Internal Amplification Control. The qPCR results corroborated the 16S rRNA and whole genome sequencing findings: VD children more often have *E. coli* in their samples compared to CS children, whereas CS children more often have *Klebsiella* spp. and *Enterococcus* spp. present in their samples than VD children (chi-square tests). Source data are provided as a Source Data file.

qPCR positive for:	0-2 RI events	3-7 RI events	p
n (%)	41	75	
Klebsiella spp. (%)	4 (9.8)	16 (21.3)	0.187
Enterococcus spp. (%)	20 (48.8)	55 (73.3)	*0.015*

Supplementary Table 7: Confirmation of association between Klebsiella spp. and Enterococcus spp. colonization at 1 week of life and more RI events later in life. Quantitative polymerase chain reaction (qPCR) analysis was performed for, amongst others, *Klebsiella* spp. and *Enterococcus* spp. on all samples of infants collected at 1 week of life (n=119). One sample was discarded from analysis due to an abnormal signal of its Internal Amplification Control. Information on RI events in the first year of life was unavailable for 2 participants. Presence of *Enterococcus* spp. was associated with more respiratory infection (RI) events in the first year of life (chi-square test). Source data are provided as a Source Data file.
