# Supplementary material for: Impact of delivery mode-associated gut microbiota dynamics on health in the first year of life
Source: Nat Commun. 2019 Nov 1;10:4997. doi: 10.1038/s41467-019-13014-7 (PMC6825150; doi:10.1038/s41467-019-13014-7)
Supplement: Supplementary file 3 — Reporting Summary [file 41467_2019_13014_MOESM3_ESM.pdf]

natureresearch

Corresponding author(s): Debby Bogaert

Last updated by author(s): Sep 13, 2019

Reporting Summary

Nature Research wishes to improve the reproducibility of the work that we publish. This form provides structure for consistency and transparency in reporting. For further information on Nature Research policies, see Authors & Referees and the Editorial Policy Checklist .

Statistics

For all statistical analyses, confirm that the following items are present in the figure legend, table legend, main text, or Methods section.

n/a	Confirmed	
☐	☒	The exact sample size (*n*) for each experimental group/condition, given as a discrete number and unit of measurement
☐	☒	A statement on whether measurements were taken from distinct samples or whether the same sample was measured repeatedly
☐	☒	The statistical test(s) used AND whether they are one- or two-sided<br>*Only common tests should be described solely by name; describe more complex techniques in the Methods section.*
☐	☒	A description of all covariates tested
☐	☒	A description of any assumptions or corrections, such as tests of normality and adjustment for multiple comparisons
☐	☒	A full description of the statistical parameters including central tendency (e.g. means) or other basic estimates (e.g. regression coefficient) AND variation (e.g. standard deviation) or associated estimates of uncertainty (e.g. confidence intervals)
☐	☒	For null hypothesis testing, the test statistic (e.g. *F*, *t*, *r*) with confidence intervals, effect sizes, degrees of freedom and *P* value noted<br>*Give P values as exact values whenever suitable.*
☒	☐	For Bayesian analysis, information on the choice of priors and Markov chain Monte Carlo settings
☒	☐	For hierarchical and complex designs, identification of the appropriate level for tests and full reporting of outcomes
☐	☒	Estimates of effect sizes (e.g. Cohen's *d*, Pearson's *r*), indicating how they were calculated

Our web collection on statistics for biologists contains articles on many of the points above.

Software and code

Policy information about availability of computer code

Data collection	Glean Study Manager™ was used to build a database for data collection. This is based on FAB™ version 2.10.1, developed by Sidekick-IT.
Data analysis	Data analysis was performed in R version 3.4.5 within R studio version 1.1.383. Packages used were phyloseq (version 1.22.3), vegan (version 2.4-5), metagenomeSeq (version 1.20.1), nlme (version 3.1-131), survival (version 2.41-3), survminer (version 0.4.3), ggplot2 (version 2.2.1) and ggpubr (version 0.2).

For manuscripts utilizing custom algorithms or software that are central to the research but not yet described in published literature, software must be made available to editors/reviewers. We strongly encourage code deposition in a community repository (e.g. GitHub). See the Nature Research guidelines for submitting code & software for further information.

Data

Policy information about availability of data

All manuscripts must include a data availability statement. This statement should provide the following information, where applicable:

- Accession codes, unique identifiers, or web links for publicly available datasets
- A list of figures that have associated raw data
- A description of any restrictions on data availability

Sequence data that support the findings of this study have been deposited in the NCBI Sequence Read Archive (SRA) database with BioProject ID PRJNA481243 [https://www.ncbi.nlm.nih.gov/bioproject/481243] and PRJNA555050 [https://www.ncbi.nlm.nih.gov/bioproject/555020]. The source data underlying Table 1, Figures 1A-C, 2, 3A-B and 4, Supplementary Tables 1 and 5-7 and Supplementary Figures 2, 5 and 6 are provided as a Source Data file.

Field-specific reporting

Please select the one below that is the best fit for your research. If you are not sure, read the appropriate sections before making your selection.

☒ Life sciences ☐ Behavioural & social sciences ☐ Ecological, evolutionary & environmental sciences

For a reference copy of the document with all sections, see nature.com/documents/nr-reporting-summary-flat.pdf

Life sciences study design

All studies must disclose on these points even when the disclosure is negative.

Sample size	The study had originally been powered based on the abundance and distribution of previously published microbiota data from infants, ensuring a power of 0.8 to detect at least significant differences in alpha and beta diversity between groups, as well as differences in abundance of the 25 most important operational taxonomical units (OTUs), taking into consideration OTUs with high and low variability and abundance and varying effect sizes. This power calculation was later verified by an online (HMP-based) tool. We initially aimed to enroll 88 infants, 44 infants per delivery mode group, allowing a drop-out of 10%. Due to uneven enrolment in both arms, approval was granted by the ethical committee to prolong enrolment to ensure sufficient caesarean section (CS) recruitment, simultaneously continuing the parallel enrolment of vaginally delivered (VD) infants to prevent seasonal/annual differences in microbiota development between the delivery mode groups. Eventually, 78 VD and 52 CS children were recruited; 10 (7.7%) dropped out after an average of 2 weeks of follow-up.
Data exclusions	No data were excluded from the analyses.
Replication	We performed confirmatory shotgun sequencing on a subset of samples to validate the major 16S-rRNA-based sequencing results. We performed qPCR for a panel of biomarker species on a large set of samples, to validate our 16S-rRNA-based sequencing results on species level. We performed both parametric and non-parametric testing to validate our findings.
Randomization	All women giving birth vaginally or by CS in the reasearch area were eligible for the study. Randomization was not applicable for this observational study.
Blinding	Blinding of investigators was not performed, because this was not relevant to our observational study.

Reporting for specific materials, systems and methods

We require information from authors about some types of materials, experimental systems and methods used in many studies. Here, indicate whether each material, system or method listed is relevant to your study. If you are not sure if a list item applies to your research, read the appropriate section before selecting a response.

Materials & experimental systems

n/a	Involved in the study	
☒	☐	Antibodies
☒	☐	Eukaryotic cell lines
☒	☐	Palaeontology
☒	☐	Animals and other organisms
☐	☒	Human research participants
☐	☒	Clinical data

Methods

n/a	Involved in the study	
☒	☐	ChIP-seq
☒	☐	Flow cytometry
☒	☐	MRI-based neuroimaging

Human research participants

Policy information about studies involving human research participants

Population characteristics	The population characteristics are detailed in the baseline table (Table 1).
Recruitment	Data and fecal samples were available from 120 children, of which 74 were VD and 46 CS infants, and their mothers, who had participated in the prospective Microbiome Utrecht Infant Study (MUIS) consisting of healthy, full term Dutch children. These 120 children had all 1) completed the initial one-year follow-up (except for two children, for whom samples were only collected until 6 months, after which they dropped out due to moving out of the area) and 2) had at least five fecal samples available from ten timepoints. Recruitment took place during pregnancy. All women giving birth vaginally or by CS were eligible for the study. After delivery, all eligible infants were enrolled in the study unless interfering criteria hindered enrolment (severe perinatal/ neonatal/maternal complications as asphyxia, resuscitation, transfer to neonatal intensive care unit, etc.; major congenital anomalies; langauge barrier; inention to move outside the research area; parents under the age of 18 years).
Ethics oversight	Ethical approval was granted by the national ethics committee in the Netherlands, METC Noord-Holland (committee on research involving human subjects, M012-015).

Note that full information on the approval of the study protocol must also be provided in the manuscript.

Clinical data

Policy information about clinical studies

All manuscripts should comply with the ICMJEguidelines for publication of clinical research and a completedCONSORT checklist must be included with all submissions.

Clinical trial registration	The MUIS study is registered in the Netherlands Trial Register under number NTR3986.
Study protocol	The full trial protocol is available per request.
Data collection	Recruitment took place on the gynaecology and obstetrics departments of the Spaarneziekenhuis in Hoofddorp and the Kennemer Gasthuis in Haarlem and at midwifery clinics in regions Groot Kennemerland and Bollenstreek. All participants were born between December 2012 and June 2014.
Outcomes	For the definition of the primary and secondary outcome measures and the assessment of these measures we refer to our detailed statistical analysis scheme (Supplementary Figure 7).
